# Impacts of the COVID-19 Pandemic on the Bereaved

**DOI:** 10.1177/10541373221151105

**Published:** 2023-01-31

**Authors:** Soraya A. Janus, Steff King, Vienna C. Lam, Gail S. Anderson

**Affiliations:** 1 School of Criminology, Centre for Forensic Research, 1763 Simon Fraser University, Burnaby, Canada

**Keywords:** COVID-19, bereavement, grief, mourning, pandemic

## Abstract

The COVID-19 pandemic has killed millions across the world in only two years. Government health restrictions aimed at preventing transmission have impacted typical mourning practices such as funeral gatherings and in-person grief support services. This research examines the potential impacts that the pandemic may have had on people's ability to grieve. We employed a mixed methods study design to ask those who have lost a loved one during this time to reflect on their mourning practices with an anonymous survey. Our results present themes of complicated grief, the uncertainty of who to blame for frustrations, and common needs requested by the bereaved to help them mourn during these unprecedented times. These findings may help inform grief support and bereavement services during current and future mass death and pandemic health concerns.

## Introduction

The novel coronavirus (COVID-19) emerged as a respiratory illness during an outbreak in Wuhan, China (Hui et al., 2020; Roberts, 2020). By March 2020, the virus had reached pandemic levels and was officially declared a global health pandemic by the World Health Organization (WHO, 2021). As of September 1, 2021, WHO confirmed over 4.5 million global deaths related to the coronavirus and over 217.5 million cases worldwide (WHO, 2021).

The mandatory restrictions enacted across British Columbia required many people to abruptly alter their normal way of life. In a short period of time, restructured death and dying practices greatly impacted the loved ones left behind. Bereavement involves a complex process of social and psychological factors, affecting each person differently but ultimately impacting personal wellness and mental health. With the increasing number of deaths during the pandemic, it is only reasonable to assess how the pandemic may have impacted grief and mourning practices, particularly amidst global and international health concerns and restrictions. Verdery and colleagues (2020) posit that there are approximately nine bereaved people for every single death, suggesting that there is a significantly high rate of bereaved across the world. Thus, we employed a mixed-method design to identify the impacts that COVID-19 may have had on the bereaved in Canada and propose recommendations that may help support those affected in the future.

## Literature Review

The bereaved often grieve in their own ways, but existing literature about the effects of previous epidemics and the early stage of COVID-19 tells us that there may be commonalities in how mass health concerns limit the grief process. Before examining the methods and outcomes of the present research, it is first important to contextualize the study with historical and modern viral outbreak impacts on public wellbeing, and the effects of complicated grief during the COVID-19 pandemic.

### Historical Accounts of Grief During Epidemics

COVID-19 is not the first global health concern impacting the bereaved. Past outbreaks of Ebola, SARS, and cholera, to name but a few, have had high death rates leaving many bereaved. Mayland and colleagues (2020) identified common themes in a review of previous pandemics on grief and bereavement including feelings of uncertainty by families and professionals about how to respond to the disease. The restrictions that followed these uncertainties often disrupted families’ connectedness to their dying loved ones and the hospital staff that cared for them, as well as affected their autonomy to visit the deceased or hold preferred death rite ceremonies. Elston and colleagues (2017) further uncovered that many families suffered long-term psychological problems following the impacts that the Ebola outbreak had on their mourning practices and grief. In past outbreaks, the priority for maintaining health protocols outweighed the personal and psychological needs of families to grieve.

### COVID-19 Vulnerability

In the context of COVID-19 and given the extreme effects of the pandemic as a whole, individuals have and are continuing to suffer various challenges. It is important to acknowledge the differential impacts COVID-19 has had on society's most vulnerable. By adding further complexities and challenges, vulnerable groups’ ability to respond and recover is greatly impacted and, without proper resources in place, can present extra challenges for the bereaved. Gaynor and Wilson (2020) discuss the breakdown of the COVID-19 virus and how it “does not see race, gender, or class yet it interacts with each of these modifiers in ways that exacerbate the existing oppressive systems that operate to maintain social hierarchy” (p. 836).

The widespread impacts of COVID-19 have been felt across the globe. Many vulnerable populations that have been impacted including (but not limited to): the elderly, people with disabilities, people with (chronic) health conditions, women, Indigenous populations, etc. The bereaved themselves also form a vulnerable group.

### Complications of Grief

Coronavirus has contributed to a variety of mental health concerns across the globe as restrictions and quarantines separate people from support networks and access to resources. The isolationist way of life during the pandemic was unprecedented (Gesi et al., 2020; Kristensen et al., 2012). People mourning loved ones were blocked from expressing and sharing their grief experiences with friends and family (Guité-Verret et al., 2021). Based on empirical findings, Vachon and her colleagues (2021) developed a definition of “pandemic grief”: neither normal nor “complicated,” pandemic grief is a hushed mourning process suspended in time, punctuated by public health measures, with little social recognition for the suffering it causes (p. 372). Grief can be influenced by a multitude of diverse factors and are likely to have become more complicated during the pandemic. Complicated grief during the time of COVID-19 found many struggling through the bereavement process, particularly when trying to find “meaning” to a loss (Wang et al., 2020; Wilson et al., 2016). The bereaved experienced suffering and guilt through absence and isolation, with the impossibility of being present at the bedside, struggling to say goodbyes, and the absence of being present at the time of death (Guité-Verret et al., 2021). Within a short period of time, COVID-19 made a long-lasting impression changing our everyday life and social habits, leaving the bereaved particularly vulnerable to traumatic grieving practices (Gesi et al., 2020). The COVID-19 pandemic will lead to a unique awareness of the value and meaning of complicated grief (Bermejo, 2020).

## Methods

This mixed methods study aimed to improve our collective understanding of how the pandemic has impacted bereavement practices. To guide this research, the primary research question is *What are the lived experiences of the bereaved during the COVID-19 pandemic in Canada?* To answer this question, we employed an exploratory mixed-method design using responses from an online survey for the bereaved.

### Data Collection

Utilizing Microsoft Forms, an anonymous survey was distributed to Canadian online bereavement forums, grief support services, and medical centers. Survey questions asked those self-identifying as bereaved during the COVID-19 pandemic to provide both non-identifying demographic information (such as age and gender) as well as to explain their mourning processes and how coronavirus may have impacted those processes. The survey was designed to provide both closed and open-ended questions so that participants could provide as little or as much information as they wished about their bereavement experiences. Data collection took place between January 1st and August 1st, 2021.

### Data Analysis

The focus of the qualitative research was to develop common themes, using a grounded theory approach, from the open-ended questions about mourning practices during the pandemic. Participants were asked 17 questions to help gain an understanding of bereavement through the lens of one's lived experiences surrounding the death and dying of a loved one during COVID-19. The process began with open coding, to assist with finding connections and relationships. To account for interrater reliability each researcher independently coded for themes, which were then compared amongst the group. Decisions were agreed upon and a consensus was made to select prevalent themes (O’Connor & Joffe, 2020). Information was fruitful and uncovered layers of personal feelings, stories, and memories. Many respondents shared very similar stories of grief and prolonged hardships. Therefore, themes and subthemes emerged from the data and were easily identifiable.

Descriptive statistics were generated to provide an overall snapshot of the respondent demographics and to provide a summary of their sentiments. Bivariate analyses were conducted to see if the participant's demographics had a statistical impact on the way they responded. Our intentions were to conduct post-hoc tests should the bivariate analyses reveal any statistically significant relationships, but this was unfortunately not possible based on the results and the limited number of respondents for certain cells.

### Ethics

Both procedural and practical ethical considerations were made throughout this study. Institutional ethics approval was obtained through Simon Fraser University's Office of Research Ethics (Study ID: 2020s0420). Adhering to the Tri-Council Policy Statement (TCPS-2) guidelines for research ethics, participants were informed of the purposes of this study before beginning the survey and could only take part by providing informed consent. The survey was designed in such a way that no identifying information was sought, though participants could add information freely in open-ended text boxes. In situations where identifying information was shared, these data were removed from further analysis. Regarding data stewardship, all data (raw or analyzed) were only accessible to the research team and stored in a secure manner. Additionally, given the emotional and psychologically demanding nature of the topic of bereavement and losing a loved one, support resources were provided at the end of the survey. Researcher emails were also provided to allow participants to ask further questions about the study.

## Results

A total of 130 responses were collected during the seven-month period (Jan 1st to Aug 1st, 2021). Of these responses, 15 were excluded because the death had occurred prior to the pandemic and an additional 17 responses were excluded based on being incomplete (as determined by answering fewer than half of the survey questions). Not all deaths being mourned were directly linked to SARs-COV-2 or COVID-19 related complications, but they were included because the conditions of the pandemic had nonetheless impacted the bereaved. The results section to follow will summarize the varying demographics of these bereaved participants as well as explore their personal struggles with complicated grief, directing blame, and asking for support.

### Respondent Demographics

The contextual differences related to pandemic restrictions differed greatly between different locations, so we took note of respondents’ primary residences. Of the 71/98 participants that shared their location, 54.1% (53) of the participants reside in British Columbia, 11.2% (11) reside in other provinces across Canada, 6.1% (6) reside in the United States, and 1% (1) reside in India (Table 1). As such, the results of this research will predominantly reflect the views of those living in Canada and in particular, residents of British Columbia.

**Table 1.** Geographic Location of Respondents (n = 71).

		Location of the Respondent			
		Frequency	Percent	Valid Percent	Cumulative Percent
Valid	British Columbia	53	54.1	74.6	74.6
	Alberta	2	2.0	2.8	77.5
	Saskatchewan	3	3.1	4.2	81.7
	Manitoba	3	3.1	4.2	85.9
	Ontario	3	3.1	4.2	90.1
	United states	6	6.1	8.5	98.6
	India	1	1.0	1.4	100.0
	Total	71	72.4	100.0	
Missing	System	27	27.6		
	Total	98	100.0		

**Figure 1.**
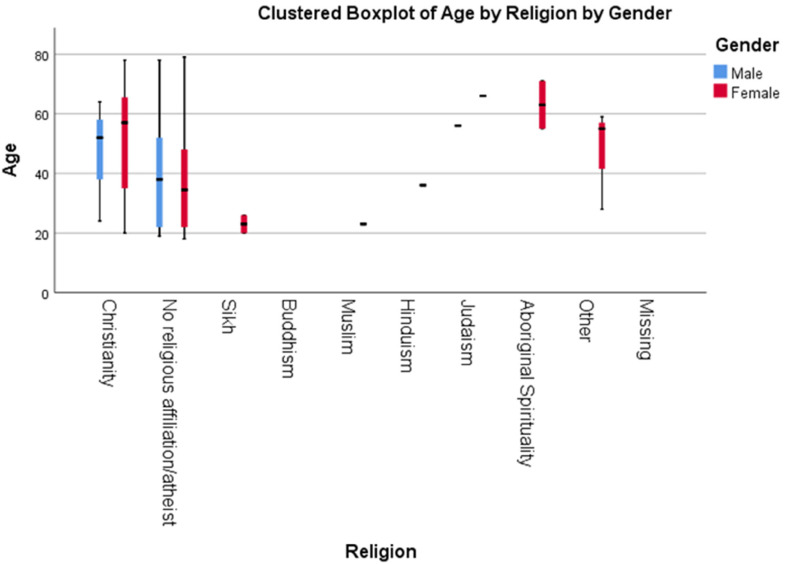
*Clustered boxplot to show the breakdown of age by religion, for both male and female respondents.* The black vertical lines show the total range, the colored bars show the range of 95% of the participants, and the black horizontal line shows the mean.

A total of 87.8% (86) of the participants self-identified as female and 12.2% (12) as male. Although the option was available, no respondent identified as gender variant. The age of the respondents ranged from 18 to 79, and were primarily Christians (41.8%, 41) or had no religious affiliation/atheist (45.9%, 45) (Figure 1). As seen in Figure 1, the clustered boxplot of the age of participants based on their religious affiliation and gender shows that most of the respondents between late 30 s to 60 s are Christian, and the younger skewing respondents reported having no religious affiliations.

Of those that responded, 75.5% (74) identified as having lost one person, whereas 23.5% (23) had two or more people die. In one case, one participant mentioned that they had lost 38 people in their immediate community. The four participants (4.1%) that lost someone in December of 2019 were included, as their mourning practices may still have been impacted. As for the other bereaved, 68.4% (67) lost someone in 2020, and 16.3% (16) in 2021.

Given that there were several open-ended questions in the survey, participants were able to choose the breadth and depth of the substance of their responses. Surprisingly, many participants provided very detailed accounts of their experiences and feelings, as if this survey provided them with a platform and opportunity to share their experiences about tragedy and death. The main thematic outcomes that emerged from these responses include: 1) Grief, 2) Assigning Blame, and 3) Needs and Expectations.

It was assumed that respondent demographics could potentially impact how people respond to a family death, but bivariate analyses revealed that age, gender, religious affiliation, number of people lost, or when the loss occurred, had no statistically significant bearing. As such, no post-hoc tests were conducted. Please note that the response rate for certain quantitatively defined variables was so low that it would not be appropriate to run further statistics. Results were also limited by the fact that most distributions were not normal (based on ± 2 kurtosis and skewedness) and there were other factors that suggest that statistical assumptions would be violated.

### Theme One: Complicated Grief

The overarching theme from the survey participants was plain and simple; grief. This theme unpacks the experiences, thoughts, and feelings of those who lost a loved one during the global health pandemic.

From 2020 into 2021, many restrictions were implemented across Canada, increasing in magnitude as cases spiked and the number of hospitalizations and patients in ICU increased. Participants identified various hardships about these restrictions including: travel restrictions, social distancing, and event gathering restrictions—especially gathering for funerals and burials. As a result of the restrictions, participants felt alone in their grief as well as a sense of surrealism or detachment from the mourning experience. Some participants said:

“Social distancing and limited interaction prevented me from being able to do a lot of the normal coping strategies my family and I had. We were unable to spend time with close family and friends in order to properly mourn our losses.”

“We had severe restrictions on visiting in hospital so we’re unable to advocate and support my mom. Current restrictions mean my dad cannot see all his children and grandchildren.”

#### Isolation

Many participants stated that they felt alone and isolated in their grief. Many missed the opportunity to say a final goodbye, be present at a loved one's bedside, mourn traditionally, or share a hug, for example, respondents stated:

“Unable to see my grandmother before she died and unable to meet together for a funeral and unable to attend the graveside service.”

“We are unable to this day to gather as a family to honour our family members who have passed away in our traditional way.”

“Not allowed to console each other.”

One participant, who lost her husband, described how the pandemic affected her in “every way”:

“Every way. Travel to visit family, them to visit me as many are out of town. In person visits for friends in town have been few & far between. People are so preoccupied with the pandemic (especially at the beginning which was right after my husband died) that the many who’d check in were very limited.”

Similarly, another participant echoed the feelings shared above, particularly how in “every way” their journey with grief (before, during, and after) was greatly impacted:

“In every way. I am just alone most of the time. Limited interaction. Friends cannot come over to support me. Hospice walking group was cancelled. I am not part of anyone's bubble. My family lives far away so cannot visit me. I cannot go out to distract myself with a play or symphony or anything I enjoy. I have not had a hug since March 2020. My dad still has not had a funeral.”

#### A Sense of “Surrealism”

Participants indicated that being constantly surrounded by COVID-19 made the stages of grief and mourning more traumatic. The continuous COVID-19 dialogue and ongoing restrictions offered constant reminders that were re-traumatizing and led to feelings of anxiety, pain, and anguish. As a result, participants explained the desire to postpone, conceal, and ignore feelings:

“Mourning has been very difficult, unable to physically connect with loved ones, knowing Covid was one of the major reasons why my son died, feeling very isolated and because we cannot finalize his death with a service makes having some closure impossible.”

“We have not been able to have any kind of service, my grandmother has not had a funeral of any type because Covid restrictions prevent that.”

The responses showed that in the face of a loss some ongoing challenges and restrictions have kept individuals, families, and communities from feeling the “normal” support, rituals, and traditions. For many there is simply no closure:

“Can't be with others. Can't bring him home. Can't run away.”

Many who have lost a loved one during the pandemic never got a chance to say goodbye or be present during the last moments. For many, they are mourning a loss that they are still trying to mentally comprehend.

### Theme Two: Assigning Blame

Assigning blame and feelings of anger towards the pandemic is one of the stand-out emerging themes from participant responses. The majority of participants provided lengthy responses expressing their feelings about a virus that has taken so much from so many. Many participants blamed the COVID-19 pandemic specifically for their feelings of personal guilt and missed opportunities.

#### Personal Guilt

One participant re-lived the tragic death of her friend and combats feelings of “immense guilt” while struggling to find ways to honor the memory of her friend:

“[…]This year, an event has been planned again (going against COVID-19 restrictions about gatherings) and I feel immensely guilty. I already carry a lot of guilt around my friend's death, as much of our friend group does, and now I have to think about this gathering as well. If I don't go, I will feel guilty about not honouring my friend with all the people he loved the most. However, if I do go I will feel guilty about putting others at risk by breaking the rules put in place for gatherings. I think no matter what it's going to be a hard day, and with COVID-19 I’m dreading it even more.”

In light of restrictions, another participant shared feelings of guilt in mourning their loved one together as a family, since only a small number of close family/friends were permitted to gather for a funeral:

“Limited number of people [were] allowed at the funeral, some family [weren't] able to attend. Limited numbers of people allowed at post-funeral services. Felt guilty for mourning together as a family in light of restrictions.”

For those who cannot or who are unable to understand the ongoing effects and restrictions associated with the pandemic, the ramifications felt by family members and close friends can be equally as traumatizing. One of the participants shared that their mother is putting a lot of blame on her, despite her mother not understanding the current situation:

“I’m taking a lot of blame from my mother that a service couldn't happen because of Covid. She doesn't understand. So, I have no one to talk to as she just gets angry at me for him passing, not me.”

As in the above example, covid-blaming can easily turn into people-blaming. The pandemic forced many people to restructure their daily lives. Due to ongoing stressors during the pandemic, many people shifted their blame away from the virus toward people—an “opportune scapegoat” to blame our stressors on those closest to us (Kirkey, 2021, para 2).

Guilt is a powerful emotion. Many people stated that they felt guilty about not being able to provide a proper goodbye/send-off for their loved one or just having a small funeral. Due to social distancing rules and ongoing restrictions, the need to blame someone after a traumatic or untimely death can be very strong—especially during a global pandemic where so many “normal” practices, rituals, and traditions were restricted or prohibited.

#### Missed Opportunities

The COVID-19 pandemic has changed the way people live and especially how they grieve. The sub-theme “missed opportunities” speaks to the impacts that restrictions have had on one's grieving process. Missed opportunities, especially in travel, left many unable to visit loved ones, attend a funeral or burial service, or in one instance intern a mother's ashes to her final resting place. A few participants shared the following:

“We have not been able to bury my mom or have a celebration due to travel restrictions and Covid as I live in BC and my family is in Ontario.”

“Was unable to take my son home and celebrate his life in the small community he was raised.”

“We couldn't have a funeral. We couldn't visit him/see his body. I had zero interaction leading up to it because of Covid.”

Through these missed opportunities, a lot of the responsibility fell to health professionals to act as a lifeline, especially for loved ones in long-term care facilities and hospitals. Quickly, everyone working in the medical system had to adopt additional supportive roles for both the patient and their families/friends. A respondent who was an ICU nurse stated that staff *“mourn for the loss of those we do not know”* as patient families were not permitted to visit at all. She added that it was *“challenging to create that therapeutic relationship with families”—“unless you are a staff person experiencing it, no one else truly understands the emotional trauma it can leave behind.”* The takeaway here is that through restrictions, opportunities were lost and grief became complicated and prolonged, impacting the bereaved but also the community at large. Overall, the majority of the participants blamed COVID for their grief, loss, and missed opportunities.

### Theme Three: Needs and Expectations

Participants were asked a final question: Do you have any last thoughts or recommendations for how public services or affiliated organizations may best address your needs? Despite the open-ended question, many participants recommended similar needs for access to grief support and outreach programs that were more sympathetic to COVID-19 struggles. Hand-in-hand with these suggestions was another prominent theme split in two polar directions: whether pandemic restrictions should be lifted for mourning practices or remain in place.

#### Support and Outreach

Grief support services are important for the wellness of the bereaved generally, whether during a pandemic or not, but for the people who lost a loved one during COVID-19, they expressed a need for specialized grief support that understood their unique concerns. One participant stated that:

“[There needs to be] more grief counselors who understand [COVID-19] and more outreach to a society that will be faced [with] grief at some points but doesn't want to hear about it.”

For reasons expressed in the previous themes, those who have grieved during the pandemic struggled to complete their usual mourning practices and expressed signs of poor mental wellness as a result. Grief support programs more attuned to how COVID-19 restrictions may impact the bereaved is a necessary tool to help those who cannot complete the grief stages while separated from loved ones, who were denied complete death rite ceremonies, or who were unable to use technological tools for virtual visitation.

Aligned with this recommendation for more specific pandemic grief support is the need for local or federal governments to make such services more accessible to the public. One participant suggested that “*There needs to be more publicly funded grief support services in Canada. Plain and simple.”* Some participants mentioned difficulty gaining access to standard support services due to location, awareness, or financial reasons. They suggested that better access to standard services, or improved awareness of local programs, may relieve the stress from families attempting to find resources for themselves while also balancing the other worries that come with the death of a loved one.

#### Provincial Health Orders Versus Grief

Alongside suggestions for better grief support came reflections on how health restrictions have impacted participant mourning. The recommendations that followed were split evenly between participants who either believed that restrictions should loosen or be lifted completely for the bereaved to grieve and those who believed restrictions are important for safety and should remain in place despite their negative impacts on the grieving process. One such participant that agreed with the former stated that:

“More needs to be done to support families who are losing loved ones during this pandemic so they can grieve easier. There needs to be more planning and understanding on [loosening] social distancing regulations during times when people are passing away.”

For those that expressed the need to lift restrictions, even only enough to allow a few more people into a funeral or hospital visitation, there was clear agreement that this should only happen in times of the death of a family member or close friend. Sentiments often reflected that the bereaved should have the right to wave restrictions temporarily during the final visitation with the dying through to the burial ceremony, all without acknowledging the potential health risk to those who may attend these events.

“I understand Covid is bad and important to take seriously. But when an individual is dying they need to be slightly more lenient with the family. We are trying to say goodbye and mentally prepare ourselves and it's hard to do that when the hospital staff are arguing with us about visitation.”

On the other side of the argument were those that acknowledged that restrictions make mourning harder but that they were also important for ensuring the health of themselves and others. One of these participants explained the following:

“I don't believe that my need to grieve “normally” [supersedes] the need to address safety concerns in regards to group gatherings during the pandemic.”

These participants commonly accepted restrictions in two manners, either by using an apathetic or opportunistic tone. Apathy came in the way of accepting the way things are and the need to move forward no matter the personal struggles: “*It is what it is and we have to deal and move on.”* The opportunistic responses provided a similar “this is the way things are” mentality but provided a positive reflection on how virtual accommodation or altered mortuary practices have made mourning just a little easier:

“I understand the restrictions put in place are needed. While it was sad to not be with family, I am still really grateful to have had the opportunity to “be” with them over Zoom, celebrating my cousin's life.”

Participant recommendations appear to differ with personal prioritizations of either immediate or future mental health and wellness. Generally, the bereaved want help from professional programs and agencies to manage their grief during these difficult times. When taking on grief themselves, however, it is a coin flip between completing original mourning practices from the beginning, despite potential health risks, or modifying or delaying practices until after the pandemic subsides, despite the potential mental health risk from incomplete grieving.

## Discussion

Not since the Spanish Flu of 1918, has the world experienced a global health crisis like COVID-19. If a death occurred during the pandemic, depending on the type(s) of restrictions in place, the bereaved may not have been able to be present at any stage of the death process, including holding a funeral, memorial, and/or burial. In the past, grief was broken into five stages (Kübler-Ross, 1969) but we now know that one's grief journey is entirely unique, unpredictable, and can vary between individuals. We know that during the COVID-19 pandemic, the grieving process has been complicated. Therefore, the bereaved may have prolonged grief, or experience what is called “complicated” or “disenfranchised” grief (Aguiar et al., 2022). The discussion to follow explores the relationships between participant themes of grief, blame, and support with context from existing literature. The section ends with a discussion of study limitations.

### Complicated and Disenfranchised Grief

According to Shear et al. (2012), a condition known as complicated grief may arise from or encompass a variety of other mental health conditions including depression and post-traumatic stress disorder (Carmassi et al., 2014, 2015; Dell’Osso et al., 2012). Our results show that people who were bereaved during COVID-19 place guilt upon themselves for not being able to say goodbye to a loved one, which is shown to be an “independent risk factor for complicated grief” (Gesi et al., 2020, p. 3). Further, respondents stated that they feel guilty for not making more of an effort to be with a loved one during the death process, despite travel restrictions and mandates at healthcare facilities. It is challenging to know just how prevalent complicated grief is but existing literature has shown that it can impact anyone and be portrayed in a variety of devastating ways (Wilson et al., 2022). It is thus imperative that a shared experience is developed to include the bereaved in end-of-life experiences, especially when circumstances have already cut them out of so much.

In 1989, Kenneth Doka first introduced the term “disenfranchised grief” and defined it as: “the process in which the loss is felt as not being openly acknowledged, socially validated, or publicly mourned” (p. xv). With so many restrictions in place, society's grieving norms have been drastically altered. Therefore, when a loss “does not accommodate these guidelines, the resultant grief remains unrecognized and undervalued and a person may feel that their ‘right to grieve’ has been denied” (Albuquerque et al., 2021, p. 2). To conform with the guidelines, the bereaved may experience a rise in “pandemic-related anxiety” and a depletion in the “emotional resources” needed to grieve a loved one (Albuquerque et al., 2021, p. 3).

Grief and loss can encompass so many things and be unique to every person. However, during the COVID-19 pandemic, individuals are experiencing psychological and physical symptoms of grief in response to the ongoing isolation and missed opportunities to be with loved ones. According to Zhai and Du (2020) as a “result of unusual prolonged and disabling grief, more individuals are at greater risk of prolonged grief disorder (PGD) in this pandemic” (p. 80). The overarching theme of grief has opened the need for further discussions and strategies to help individuals cope with their unique set of tragic circumstances.

### Blame During the Pandemic

The unique circumstances and stressors that continue to come out of the COVID-19 pandemic highlight blame and stress spillover. During the beginning of the pandemic, increased levels of stress and anxiety plagued many people and increased tensions in relationships (Pietromonaco & Overall, 2021). As previously discussed, assigning blame was one prominent theme outlined by participants. The blaming added stress, internal conflict, and external conflict with family, friends, and the community. Perhaps not surprisingly, participants described their constant internal conflict with the health orders and restrictions, commonly analyzing their own actions but also feeling pressures and judgments from others. An ongoing cycle of blaming emerged, and still continues.

A study conducted by Neff and colleagues (2022) on COVID-19 blaming confirmed that, on average, individuals were more likely to blame the pandemic than they were to blame themselves or their partners for their problems. As the pandemic evolved and new waves emerged with variants of concern, the overwhelming amount of information (including an abundance of unreliable information) contributed to an incredible amount of loss. The health crisis has been described as an “infodemic” by WHO, amplification of social media and other platforms with increasing amounts of misinformation have soared, among other growing frustrations, blame toward COVID-19 was at the forefront along with many other factors of loneliness, guilt, and loss. Similar findings were shown internationally, specifically to Portuguese bereaved adults (Aguiar et al., 2022). To blame the pandemic is opportune, easy, and a harmless scapegoat for one's feelings and emotions.

### Recommendations and Mitigation Strategies

Interestingly, participants’ recommendations were similar to those proposed in the literature. Mayland and colleagues (2020) report that many existing studies suggest that there are a variety of ways to mitigate grief during times of health crises. This includes creating and maintaining social connections and improving communication between family members. This reflects participant acknowledgments that social distancing and travel restrictions played a hand in their incomplete grieving as they were prevented from visiting the dying or from physically supporting one another after the death of a loved one. Families reported their attempts to work around these restrictions, from virtual funerals to limiting the number of people at a ceremony, but even with these accommodations they still expressed feelings of anger, guilt, depression, and isolation due to their inability to mourn with others physically. Aguiar and her colleagues (2022) reported similar findings highlighting the devastating effects of missed opportunities, unable to express their grief openly and in traditional settings (p. 6). The problem here, however, is that restrictions prioritize decreasing the risk of exposure to COVID-19 over negative impacts on personal mourning—something half of the respondents accepted. This complicates creating future recommendations for how to mitigate the negative impacts that restrictions may have on the bereaved.

Downar and Kekewich (2021) suggest that family actions may minimize the effectiveness of gathering size restrictions to prevent COVID-19 transmission. For families adamant about seeing loved ones, either at their death bed or at funerals, they may still find ways around the restrictions that diminish their overall effect. Cohabiting families may switch out the number of permitted people into a hospital room or funeral service and later transmit to each other outside of the venue. Downar and Kekewich suggest, in these instances, that it would be more effective to ensure the appropriate use of personal protective equipment for those visiting and routine cleaning of visited areas. This would allow families to visit and physically support one another in a way that may decrease feelings of isolation or incomplete grieving later on. That said, Haug and colleagues (2020) still report that small gathering capacity remains one of the most effective ways to lessen the chance of COVID-19 transmission.

Inclusive grief support and professional staff communication with families may be the best option for addressing restriction and grief processing challenges. Professional staff who interact with the bereaved (e.g., hospital or mortuary) would do well to explain to families why restrictions are in place and their long-term benefits to personal health. They should also provide alternative means for families to interact with one another (e.g., assisted Zoom conferencing or dedicated family visitation rooms) to combat feelings of isolation and increase familial support. Otani and colleagues (2017) posit that complicated grief processes are less associated with the inability to be physically present at the time of death, rather it is more likely to do with the inability of families to talk with one another. While not every person grieves the same, nor is affected by COVID-19 and its restrictions in the same way, improving personal communication and support during the grieving process may have some benefit to a person's overall mental health and wellness.

### Limitations

The majority of the participants in this study came from Canada which may reflect mourning practices, religious beliefs, and personal values that are not shared in non-Western countries. While the thematic outcomes may not be widely generalizable, they do provide a snapshot of the experiences and impacts that COVID-19 has had on bereaved people and can inform general better practices for future crises. There were too few cases to conduct more sophisticated statistical analyses. With greater statistical power, more generalizations could be made as to the relationship of sentiments, and whether those opinions held would be statistically tied to respondent demographics.

## Conclusion

The world has been living with COVID-19 for nearly two years. Before 2020, most people did not wear a mask, socially distance, or even imagine that stay-at-home orders would be imposed or that airports would be ghost towns. The world came to a standstill. Now in late 2021, the world is still rallying against case surges and encouraging the populace to be vaccinated. Yet, in the midst of it all, the disease is still claiming thousands of lives a day and people are continuing to deal with the aftermath of these deaths. There are guidelines available to the public for how to protect oneself from contracting COVID-19 but there is little offered to help the bereaved grieve or cope with these unprecedented times. The pandemic mourners do not know how to feel, whether guilty or surreal, they do not know who to blame for their feelings, be it health restrictions or professional staff, and they do not know where or to whom to turn for support.

The journey of grief looks and feels different for everyone with so many other factors adding elements of stress and anxiety. Therefore, it is critical that the bereaved feel heard and supported—COVID-19 has undoubtedly changed the grieving process for many millions of people around the world. This pandemic has shown that we are all vulnerable and have vulnerabilities.
